# A Case of Rare Complication of Inguinal Parietoplasty according to Lichtenstein: Entero Cutaneous Fistula

**DOI:** 10.1155/2018/3592738

**Published:** 2018-01-17

**Authors:** K. B. Kouakou, K. I. Anzoua, M. Traore, B. K. I. Leh, A. B. N'Dri, A. S. Ekra, B. A. Kouakou, R. Lebeau, B. Diané

**Affiliations:** ^1^Department of General and Digestive Surgery, University Hospital of Bouaké, Bouaké, Côte d'Ivoire; ^2^Surgery Emergency Department, University Hospital of Bouaké, Bouaké, Côte d'Ivoire

## Abstract

Lichtenstein intervention is currently the classic model of the regulated treatment of inguinal hernias by direct local approach. This “tension-free” technique satisfies both patients and practitioners. However, it does not often evade severe complications of parietal surgery. The authors report their treatment experience in rural Africa of a late enterocutaneous fistula which aggravated an inguinal hernia repair according to the Lichtenstein procedure. Physiopathology, diagnosis, and treatment of that disease are analyzed in the light of literature.

## 1. Introduction

Enterocutaneous fistula due to prosthetic parietal surgery is unusual [1]. Its estimated rate ranges from 0.3 to 3.5% [2]. The one that results from inguinal prosthetic implantation is even rarer [3]. The difficulty in its diagnosis, lately after the placement of the parietal prosthesis, and its often complex treatment and morbidity make enterocutaneous fistula one of the most dreaded parietal surgeries [4]. We report our experience in the management of a late enterocutaneous fistula worsening an inguinal hernia repair under the Lichtenstein procedure. An analysis of the physiopathology, risk factors, diagnostic elements, and the treatment of this disease is carried out in light of literature.

## 2. Observation

**Figure 1:**
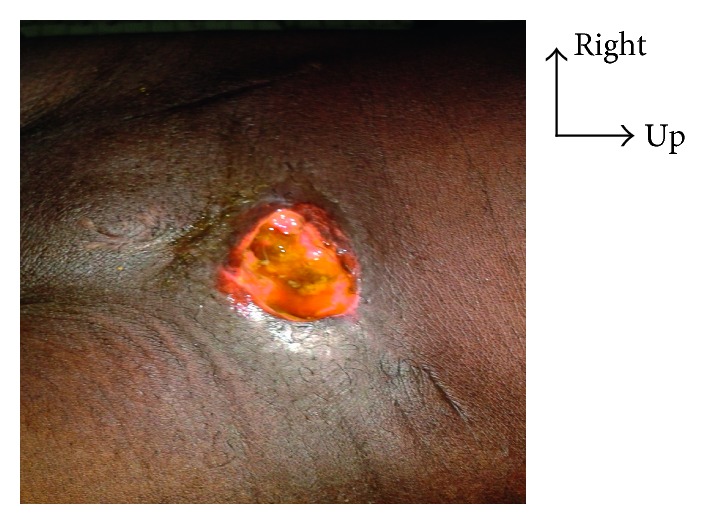
Flow of stools through a left inguinal tumefaction facing wound.

**Figure 2:**
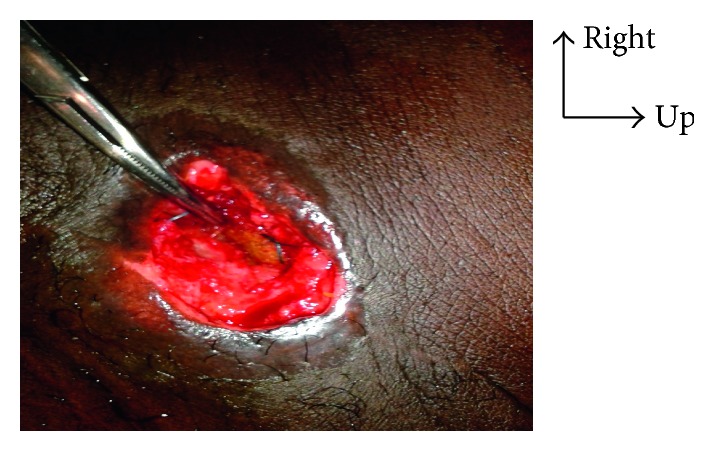
Parietal prosthesis visible through the wound.

**Figure 3:**
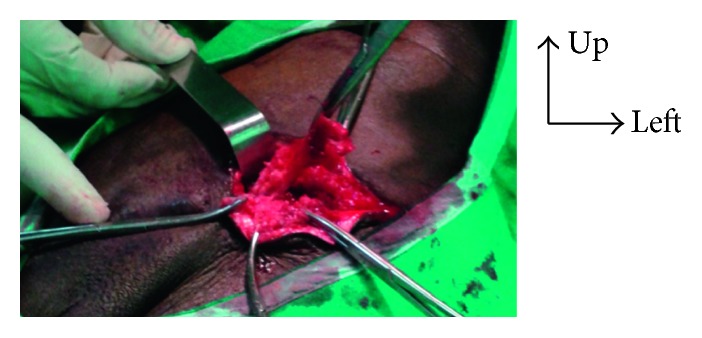
Ablation of the prosthesis via the inguinal channel.

**Figure 4:**
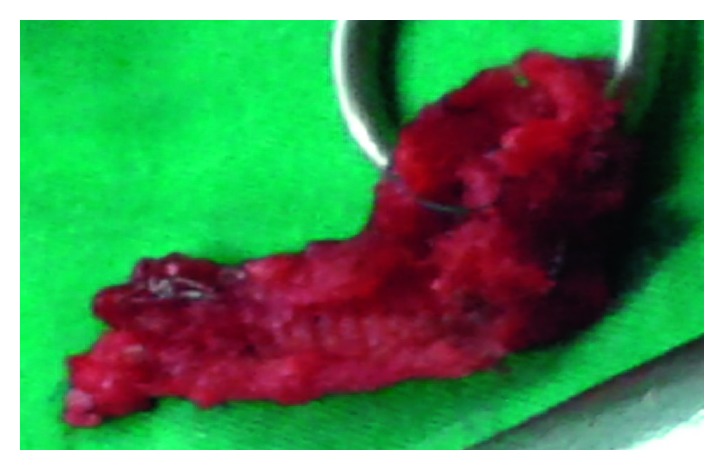
Parietal prosthesis after total ablation.

**Figure 5:**
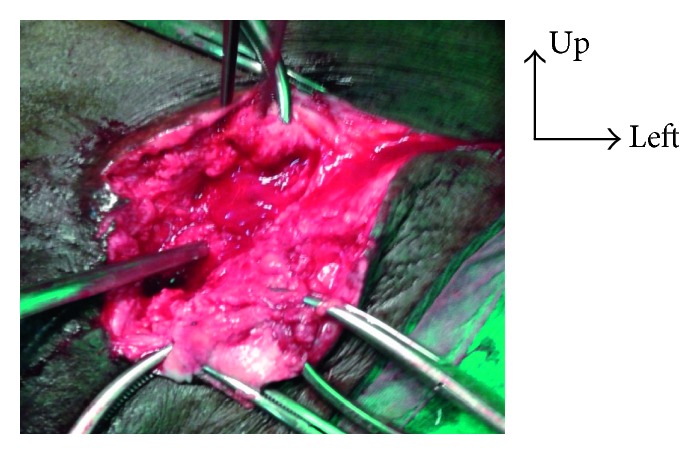
Ileal lumen after total ablation of the prosthesis.

**Figure 6:**
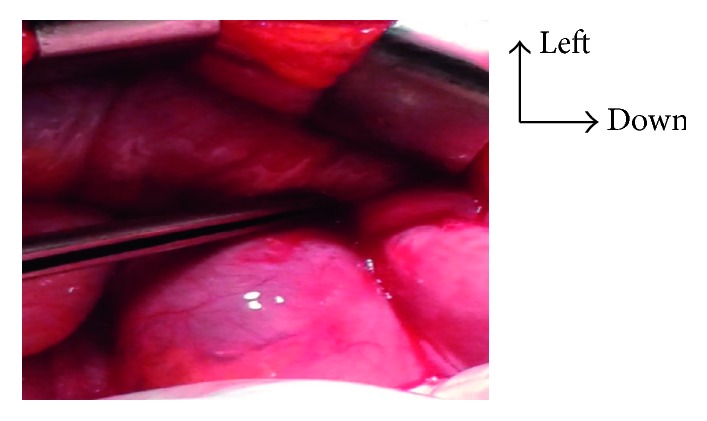
Adhesion of the ilium to the left inguinal orifice.

**Figure 7:**
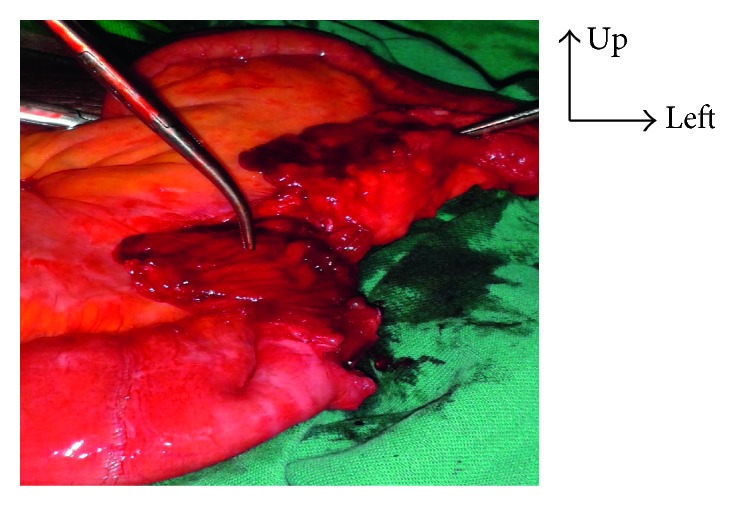
Exposure of the digestive fistula after median laparotomy.

Mr. CK, a 55-year-old farmer, was admitted to surgical emergencies at Bouaké University Hospital in September 2015 for a painful, fluctuating right inguinal swelling that has been evolving for a week. Chronic abdominal pain and unassessed fever were the associated functional signs. Intestinal transit was preserved. His surgical history is marked by a bilateral prosthetic inguinal hernia treatment under the Lichtenstein procedure to treat a left inguinoscrotal hernia and right inguinal hernia. This intervention was carried out a year ago in a private hospital. Fitted prostheses were made of Prolene® type polypropylene (size 7 × 15; Ethicon). Fixing was made with a 2-0 nonresorbable thread. The patient's initial physical examination revealed a moderate infectious facies and a preserved general condition. The left inguinal displayed through a wound the prosthetic material within a fecaloid gathering (Figures 1-2). The right inguinal zone was normal. Other hernial orifices were free. There was nothing special about the examination of the rest of the abdomen as well as other systems. Accordingly, there was no sign of severe peritonitis. Biological examinations revealed a hyperleucocytosis with 11874 leukocytes/ml, predominantly polynuclear neutrophils, and a normal level of serum albumin at 38 g/l. The diagnosis of an enterocutaneous fistula on an inguinal parietal prosthetic implant was retained. No radiological examination aimed at diagnosis was necessary. In emergency, the surgical treatment consisted in removing the prosthesis by inguinal approach followed by a manual end-to-end ileoileal anastomosis resection by median laparotomy (Figures 3–7). The inguinal approach was not completely closed due to suppuration. Surgery outcomes were simple; Mr. CK was allowed to leave hospital after 8 days of stay. Left hernial treatment by herniorraphy was performed 6 months later under Bassini procedure, after complete healing of the wound and without any inflammatory phenomenon; the patient gave an outright refusal to a new prosthetic treatment. The results of the latter intervention were also simple. Reviewed 12 months after this herniorraphy, Mr. CK presented no complications.

## 3. Discussion

Prosthetic implants have, to date, revolutionized parietal surgery, especially that of inguinal hernia [5]. Good tolerance and improvement of those implants' quality offer both the practitioner and patient a better comfort. Among the vast number of techniques currently popularized, Lichtenstein's intervention remains the gold standard of the inguinal approach [6]. This is due to its technical simplicity and easy reproducibility [7]. The advantages of this “tension-free” intervention, for the patient, are indisputable and hence the patient's satisfaction [8]. Complications of hernia surgery such as postoperative pain, urination disorders, and subcutaneous gathering (seroma and hematoma) are better and better controlled [6, 9]. These advantages make Lichtenstein intervention one of the first to be performed as an outpatient setting [8].

Late complications of prosthetic parietoplasty are chronic pain, surgical site and prosthesis infections, recurrence, bowel occlusion by adhesion, and prosthetic disinsertion [2, 8]. Enterocutaneous fistula after a parietoplasty under Lichtenstein procedure appears as a rare late complication. Its estimated frequency ranges from 0.3 to 3.5% [2]. This rarity is widely mentioned in the literature. Morin et al. [4], in 2001, reported only 8 cases of late digestive fistulas on parietal prosthesis in 12 years, including only one case after an inguinal hernia repair. Similarly, Szitkar et al. [2] in 2010, described only 3 cases of prosthetic migration including one case after an inguinal hernia repair. As far as we know, the observation we report is the first case described in the Ivorian scientific literature.

The physiopathology of the occurrence of this pathology is not clearly established. It may be the outcome of a progressive evolution process and hence the long free interval [10]. Owing to an incomplete closure or a breach of the peritoneum, the prosthesis is in direct contact with neighboring viscera. Adhesion, followed by parietal erosion and abscess which follow it, constitutes the source of the fistula, whose path can be externalized to the skin [4]. When the prosthesis fitting is defective or absent, the prosthesis can migrate, be in contact with the viscera, and be inserted into hollow viscus thus injured [2].

The clinical picture is polymorphic and depends on the injured organ [3]. This may explain difficulties and delays in diagnosis. It may be limited to inguinal or abdominal discomfort or pain associated with fever. It is the chronic or recurring character of these signs, and the appearance of an abscess fistulated to the wall, associated with the history of inguinal prosthetic hernia repair, that will make the diagnosis be evoked [2]. This is the case of our patient who had a fistulized inguinal abscess leaving of stools leaking with exposure of the parietal prosthesis. The clinical picture may, however, be more severe, underlining the severity of this disease. It may be a febrile occlusion by the migration of the prosthesis into the bowel lumen [11] or a generalized peritonitis by fistulization in free peritoneum associated with a septic shock [3]. A cataclysmic hemorrhagic shock clinical picture by erosion of an artery in contact with the prosthesis has also been described [4]. Various clinical presentations of this disease highlight the importance of medical imaging in the diagnosis of this pathology. MRI may be the most efficient examination. Instead, it is not always available in emergency. The multidetector scanner would be, in everyday practice, the best morphological examination for diagnostic purposes [2, 12].

In contrast to the diversity of their clinical pictures, enterocutaneous fistulas have similar characteristics. Their occurrence delay is long. This period varies from 1 to 15 years according to the authors [1, 4]. In our observation, the ileocutaneous fistula occurred one year after the placement of the parietal prosthesis by the Lichtenstein procedure. In addition, the infected postoperative seroma or hematoma and the pre- or postoperative parietal infection would constitute the bed of the stall and the migration of the prosthesis and the fistula [3]. These early postoperative complications were absent in our observation. Risk factors in the literature are the nature of the prosthesis, the seat of its implantation and its fixation or not during implantation. Multifibrillary prostheses in polyester or dacron would significantly increase the number of migrations and fistulas [4]. We do not use them in our service. Only polypropylene prostheses such as Marlex® and Prolene are used in our practice. Regarding the seat of implantation of the prosthesis, it seems established that the direct contact between the prosthesis and the viscera is a risk factor of fistula occurrence. The issue remains, however, always mentioned in the literature [3, 4, 10, 13]. In our observation, the adherence of the prosthesis to the ileum seems, to us, the most plausible hypothesis explaining the occurrence of enterocutaneous fistula.

The surgical treatment remains classical in literature. And it recommends full removal of the prosthesis [4].

## 4. Conclusion

Enterocutaneous fistula complicating prosthetic parietoplasty by the Lichtenstein procedure is rare. This is a complication that can be serious. Its pathophysiology and risk factors are not clearly established in the literature. Careful investigation of the patient's history of hernia repair and medical imaging allow for diagnosis. From our observation, it seems to us lawful to suggest as prevention means a parietal reinforcement before the implantation of the prosthesis, in the treatment of inguinal hernias presenting a great parietal weakness.

## References

[1] Hamy A., Paineau J., Savigny J. L., Vasse N., Visset J. (1997). Sigmoid perforation, an exceptional late complication of peritoneal prosthesis for treatment of inguinal hernia. *International Surgery*.

[2] Szitkar B., Yzet T., Auquier M. A. (2010). Complications tardives de la chirurgie pariétale abdominale: à propos de trois cas de migration de prothèse dans un organe creux. *Journal de Radiologie*.

[3] Pautrat K., Scotto B., Machet M. C., Huten N., De Calan L. (2004). Migration intraluminale colique d’une prothèse de cure de hernie inguinale. *Journal de Chirurgie*.

[4] Morin B., Bonnamy C., Maurel J., Samama G., Gignoux M. (2001). Fistules intestinales après implantation de prothèse pariétale abdominale. *Annales de Chirurgie*.

[5] Friis E., Lindahl F. (1996). The tension-free hernioplasty in a randomized trial. *American Journal of Surgery*.

[6] Pélissier E., Palot J.-P., Ngo P. (2007). Traitement chirurgical des hernies inguinales par voie inguinale. *Techniques chirurgicales-Appareil digestif*.

[7] Lichtenstein I. L., Shulman A. G., Amid P. K., Montllor M. M. (1989). The tension-free hernioplasty. *American Journal of Surgery*.

[8] Kraft K., Mariette C., Sauvanet A. (2011). Indications de la chirurgie digestive et endocrinienne pratiquée en ambulatoire chez l’adulte. *Journal de Chirurgie Viscérale*.

[9] Amid P. K. (2004). Lichtenstein tension-free hernioplasty: its inception, evolution and principles. *Hernia*.

[10] Leber G. E., Garb J. L., Alexander A. I., Reed W. P. (1998). Long-term complications associated with prosthetic repair of incisional hernias. *Archives of Surgery*.

[11] Ferrone R., Scarone P. C., Natalini G. (2003). Late complication of open inguinal hernia repair : small bowel obstruction caused by intraperitoneal mesh migration. *Hernia*.

[12] Tonolini M. (2016). Multidetector CT of expected findings and complications after contemporary inguinal hernia repair surgery. *Diagnostic and Interventional Radiology*.

[13] Vrijland W. W., Jeekel J., Steyerberg E. W., Den Hoed P. T., Bonjer H. J. (2000). Intraperitoneal polypropylene mesh repair of incisional hernia is not associated with enterocutaneous fistula. *British Journal of Surgery*.

